# Predicting pathological complete response (pCR) after stereotactic ablative radiation therapy (SABR) of lung cancer using quantitative dynamic [^18^F]FDG PET and CT perfusion: a prospective exploratory clinical study

**DOI:** 10.1186/s13014-021-01747-z

**Published:** 2021-01-13

**Authors:** Dae-Myoung Yang, David A. Palma, Keith Kwan, Alexander V. Louie, Richard Malthaner, Dalilah Fortin, George B. Rodrigues, Brian P. Yaremko, Joanna Laba, Stewart Gaede, Andrew Warner, Richard Inculet, Ting-Yim Lee

**Affiliations:** 1 Department of Medical Biophysics, Schulich School of Medicine and Dentistry, University of Western Ontario, 1151 Richmond St N, London, ON N6A 5C1 Canada; 2 Robarts Research Institute, University of Western Ontario, 1151 Richmond St N, London, ON N6A 3K7 Canada; 3 Lawson Imaging Research Program, Lawson Health Research Institute, 268 Grosvenor St, London, ON N6A 4V2 Canada; 4 Department of Oncology, Schulich School of Medicine and Dentistry, University of Western Ontario, 800 Commissioners Rd E, London, ON N6A 5W9 Canada; 5 Department of Radiation Oncology, London Regional Cancer Program, 800 Commissioners Rd E, London, ON N6A 5W9 Canada; 6 Pathology and Laboratory Medicine, London Health Sciences Centre, 800 Commissioners Rd E, London, ON N6A 5W9 Canada; 7 Department of Radiation Oncology, Sunnybrook Health Sciences Centre, 2075 Bayview Ave, Toronto, ON M4N 3M5 Canada; 8 Department of Surgery, Division of Thoracic Surgery, London Health Sciences Centre, 800 Commissioners Rd E, London, ON N6A 5W9 Canada

**Keywords:** Stereotactic ablative radiation therapy (SABR), Non-small cell lung cancer (NSCLC), Pathologic complete response (pCR), Dynamic positron emission tomography (PET), [^18^F]FDG, CT perfusion, Kinetic analysis

## Abstract

**Background:**

Stereotactic ablative radiation therapy (SABR) is effective in treating inoperable stage I non-small cell lung cancer (NSCLC), but imaging assessment of response after SABR is difficult. This prospective study aimed to develop a predictive model for true pathologic complete response (pCR) to SABR using imaging-based biomarkers from dynamic [^18^F]FDG-PET and CT Perfusion (CTP).

**Methods:**

Twenty-six patients with early-stage NSCLC treated with SABR followed by surgical resection were included, as a pre-specified secondary analysis of a larger study. Dynamic [^18^F]FDG-PET and CTP were performed pre-SABR and 8-week post. Dynamic [^18^F]FDG-PET provided maximum and mean standardized uptake value (SUV) and kinetic parameters estimated using a previously developed flow-modified two-tissue compartment model while CTP measured blood flow, blood volume and vessel permeability surface product. Recursive partitioning analysis (RPA) was used to establish a predictive model with the measured PET and CTP imaging biomarkers for predicting pCR. The model was compared to current RECIST (Response Evaluation Criteria in Solid Tumours version 1.1) and PERCIST (PET Response Criteria in Solid Tumours version 1.0) criteria.

**Results:**

RPA identified three response groups based on tumour blood volume before SABR (BV_pre-SABR_) and change in SUV_max_ (ΔSUV_max_), the thresholds being BV_pre-SABR_ = 9.3 mL/100 g and ΔSUV_max_ = − 48.9%. The highest true pCR rate of 92% was observed in the group with BV_pre-SABR_ < 9.3 mL/100 g and ΔSUV_max_ < − 48.9% after SABR while the worst was observed in the group with BV_pre-SABR_ ≥ 9.3 mL/100 g (0%). RPA model achieved excellent pCR prediction (Concordance: 0.92; *P* = 0.03). RECIST and PERCIST showed poor pCR prediction (Concordance: 0.54 and 0.58, respectively).

**Conclusions:**

In this study, we developed a predictive model based on dynamic [^18^F]FDG-PET and CT Perfusion imaging that was significantly better than RECIST and PERCIST criteria to predict pCR of NSCLC to SABR. The model used BV_pre-SABR_ and ΔSUV_max_ which correlates to tumour microvessel density and cell proliferation, respectively and warrants validation with larger sample size studies.

***Trial registration*:**

MISSILE-NSCLC, NCT02136355 (ClinicalTrials.gov). Registered May 8, 2014, https://clinicaltrials.gov/ct2/show/NCT02136355

## Background

Stereotactic ablative radiation therapy (SABR) is effective in treating inoperable stage I non-small cell lung cancer (NSCLC) [1]. The majority of studies of using SABR for this indication report a 3-year local control rate without surgery of approximately 90%, based on imaging [2]. However, tumour response after SABR is difficult to evaluate using current clinical evaluation guidelines, such as RECIST (Response Evaluation Criteria in Solid Tumours version 1.1) and PERCIST (PET Response Criteria in Solid Tumours version 1.0) guidelines.

After SABR, more than 50% of patients can have lung density changes on computed tomography (CT) imaging which could be due to radiation-induced lung injury (RILI) or tumour recurrence [3]. However, these changes do not have distinctive patterns that easily differentiate between RILI and recurrence. Moreover, the inflammatory RILI response shows a high fluorine-18 fluorodeoxyglucose ([^18^F]FDG) standardized uptake value (SUV) on positron emission tomography (PET) imaging, implying a hypermetabolic state as in tumour recurrence [4]. Because RILI and recurrence can have similar size, morphology and [^18^F]FDG uptake [5, 6], differentiating these entities based on post-SABR CT and PET according to RECIST and PERCIST criteria can be difficult.

CT Perfusion (CTP) and dynamic [^18^F]FDG-PET (as opposed to static SUV measurement taken at one time point) can be used to understand tissue hemodynamics (blood flow (delivery) and permeation of the endothelial barrier) and glucose metabolism, respectively. The two modalities have a practical advantage that they can be conveniently combined together in a single study session on a PET/CT scanner. Evaluation of perfusion, blood volume, and glucose uptake rate could be more sensitive than either RECIST or PERSIST at monitoring the response to SABR. The purpose of the present study was to evaluate whether the imaging biomarkers from CTP and dynamic [^18^F]FDG-PET could predict the true pathologic complete response (pCR) of NSCLC to SABR. To our knowledge, this is the first prospective study evaluating imaging-based biomarkers to predict pCR after SABR treatment with histopathological study on the explanted tumour as the gold standard.

## Methods

### Patient characteristics

This analysis was a correlative study within a phase II clinical trial (NCT02136355; MISSILE-NSCLC), and the primary analysis and full protocol have been published previously [7]. The phase II study evaluated the combination of SABR followed by surgery in the treatment of early-stage (T1 or T2a) NSCLC. The study was approved by the Institutional Research Ethics Board. All participants in this study provided written informed consent. Eligible patients aged 18 or older had histologically confirmed early-stage NSCLC (≤ 5 cm), no evidence of nodal or distant metastases (N0, M0), Eastern Cooperative Oncology Group (ECOG) status 0–2, life expectancy greater than 6 months and predicted post-operative forced expiratory volume in 1 s (FEV_1_) of 30% or greater. Exclusion criteria included severe medical comorbidities or other contraindications to radiation therapy or surgery, prior history of lung cancer within 5 years, prior thoracic radiation at any time, and allergy to CT contrast. Pregnant or lactating women were also excluded.

### Study protocol

After enrollment in this study, participants underwent the pre-SABR imaging session consisting of dynamic [^18^F]FDG-PET and CTP imaging in this order with a hybrid PET/CT scanner. At 8-week post-SABR (8-week after last fraction), participants were again imaged as in the pre-treatment session. Finally, at 10-week post-SABR, the tumour was resected.

### Dynamic [^18^F]FDG-PET and CT perfusion (CTP) imaging acquisition and imaging biomarker analysis

Dynamic [^18^F]FDG-PET was acquired on a Discovery VCT (GE Healthcare, Waukesha, WI, USA) PET/CT scanner. Prior to the dynamic PET scan, a CT localization scan for attenuation correction was obtained with patients lying supine on the patient couch. For the dynamic PET scan a bolus injection of [^18^F]FDG at a dosage of 5 MBq/kg was given, with the patient in the same position as the CT scan. Simultaneous with the injection, while the patient was breathing quietly, images covering the primary tumour and pulmonary artery were acquired for 60 min (min) with a variable frame length of 5 s (s) (6 frames), 10 s (6 frames), 20 s (3 frames), 30 s (5 frames), 60 s (5 frames), 150 s (8 frames), and 300 s (6 frames). The PET images were reconstructed using 2D-OSEM (ordered subset expectation maximization) method with a pixel size of 5.47 mm (700 mm field of view (FOV) and 128 × 128 matrix).

The CTP scan was performed immediately after the dynamic [^18^F]FDG-PET PET scan without moving the patients and also under quiet breathing. The scan was acquired over 3 min using a shuttle mode where two contiguous 4 cm sections of the thorax covering the primary tumour and the pulmonary artery, identified from the CT localization scan, were alternately scanned starting 6 s before a bolus injection of contrast agent (Isovue 370, Bracco Diagnostic Inc., NJ, USA) at a rate of 3 mL/s and a dosage of 0.7 mL/kg into an antecubital vein. The CTP images were acquired using 32 × 1.25 mm slices, 120 kVp and 50 mAs at intervals of 2.8 s for first 1 min and then every 15 s for the next 2 min. The pixel size of the CTP images were 0.7 mm (360 mm FOV and 512 × 512 matrix). The acquired free-breathing images were registered using non-rigid image registration (GE Healthcare) to minimize misregistration before generating the CTP functional maps.

From the dynamic [^18^F]FDG-PET data, kinetic parameters—K_1_ (influx rate constant) in mL/min/g, k_2_ (efflux rate constant) in min^−1^, k_3_ (binding rate constant) in min^−1^, k_4_ (dissociation rate constant) in min^−1^, K_i_ = K_1_k_3_/(k_2_ + k_3_ + k_4_) (net uptake/metabolic rate constant) in mL/min/g and DV = K_1_/k_2_(1 + k_3_/k_4_) (distribution volume) in mL/g were estimated using a previously developed flow-modified two-tissue compartment model [8] to account for blood flow delivery and birdirectional permeation of the blood-tumour barrier by [^18^F]FDG. In addition to the kinetic analysis, the last six dynamic PET images equivalent to 30 min of acquisition starting at 30 min post-injection were averaged together for SUV_max_ and SUV_mean_ measurements. Commercial software (CT Perfusion, GE Healthcare) was used to generate functional maps, including average, blood flow (BF) in mL/min/100 g, blood volume (BV) in mL/100 g, mean transit time (MTT) in seconds and vessel permeability surface product (PS) in mL/min/100 g, from the CTP imaging.

The tumour volume was manually segmented from the CTP average map using both the lung and mediastinal window for display. CT and PET tumour image biomarker values were obtained from defined CT tumour volume after CT functional maps and PET images were co-registered using the CT average map and PET SUV map with 3D-Slicer (www.slicer.org).

RECIST and PERCIST measurements were done on the CT average and SUV map.

### Stereotactic ablative radiation therapy, surgery and determination of pCR status

SABR was delivered using a risk-adapted method, with the dose and the number of fractions dependent on the size and location of the tumour. 54 Gy in 3 fractions were delivered to tumours ≤ 3 cm and surrounded by lung parenchyma; 55 Gy in 5 fractions to tumours abutting the chest wall or > 3 cm; and 60 Gy in 8 fractions to tumours within 2 cm of the mediastinum or brachial plexus [9, 10]. Individual fractions were delivered every second day, on weekdays. All patients underwent 4D planning CT simulation. Respiratory gating was considered in cases where motion was > 7 mm in any direction. The detail protocol of SABR and surgery have been described in the original publication [7].

Surgery, either lobectomy or sublobar resection, was performed at our high-volume tertiary center after the 2nd set of imaging, at 10 ± 2 weeks following SABR, to allow sufficient time for a pathological response. The at-risk hilar and mediastinal nodes were also sampled at the time of resection. The resected tumour was oriented by the surgeon to its in-vivo position and submitted for histopathology. The pCR status of the primary tumour was determined by the pathologist based on standard hematoxylin and eosin staining criteria, as described in the original publication [7].

### Statistical analysis

Recursive partitioning analysis (RPA) using decision trees was performed to create a predictive model of pCR status of patients. A minimum number of 5 observations in a node were required to enable further splitting, followed by trimming of less important downstream branches as needed. The performance of the RPA model, RECIST complete response (CR)-partial response (PR) and PERCIST complete metabolic response (CMR)-partial metabolic response (PMR) criteria in predicting pCR was compared qualitatively using sensitivity, specificity, positive predictive value (PPV), and negative predictive value (NPV) and DeLong test of concordance (C-statistic or area under the receiver operating characteristic curves (ROC)) for statistical significance. Statistical analyses were performed using SAS version 9.4 software (SAS Institute, Cary, NC, USA) and the R language for statistical computing version 3.5.0 (open source, www.r-project.org), using two-sided statistical testing at the 0.05 significance level.

## Results

**Fig. 1:**
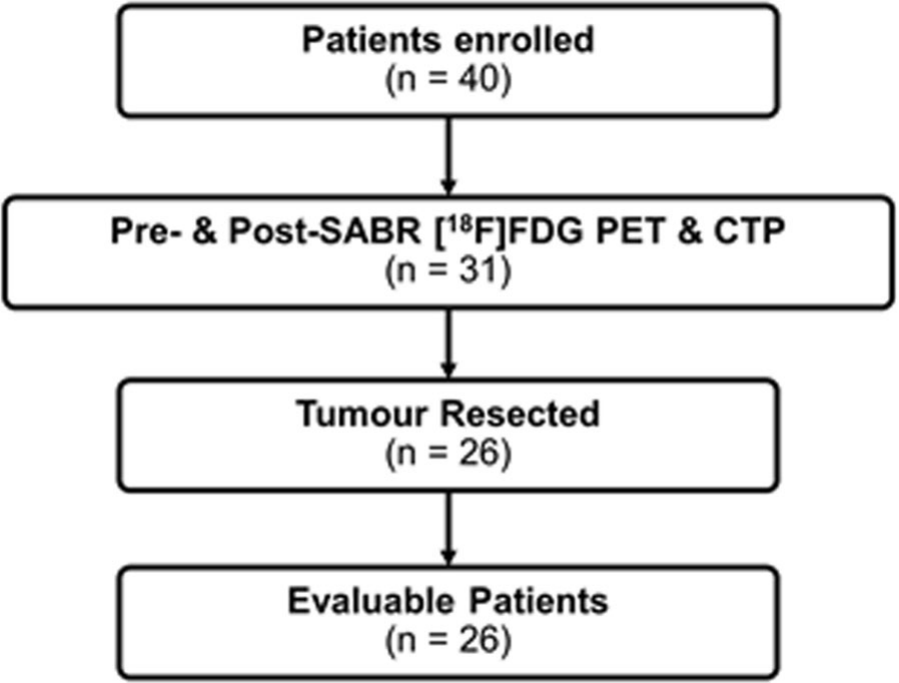
Summary of patient enrollment. *SABR* stereotactic ablative radiation therapy, *FDG* fluorodeoxyglucose, *PET* positron emission tomography, *CTP* computed tomography perfusion

Between September 2014 and September 2017, 40 patients were enrolled in this study, of which 35 were evaluable for the primary endpoint, as described in the original paper [7]. Of those 35, 26 patients completed both imaging sessions and were available to be analyzed. The other 9 declined or was unavailable for one or both of the imaging sessions. Patient enrollment is summarized in Fig. 1. Table 1 shows the clinical-pathologic characteristics of the patients. There were 13 male and 13 female patients in this sub-study. Of the 26 patients included herein, 13 patients had RILI and all had a pCR, and 13 patients had residual disease.

**Table 1:** Baseline tumour, patient and treatment characteristics for all patients (n = 26)

Characteristic	All patients (n = 26)
Age at registration—median (min, max)	68.7 (43.5, 82.9)
*Gender—n(%)*	
Male	13 (50.0)
Female	13 (50.0)
*Primary lung location—n(%)*	
Left upper lobe	2 (7.7)
Left lower lobe	3 (11.5)
Right upper lobe	13 (50.0)
Right middle lobe	4 (15.4)
Right lower lobe	4 (15.4)
Pre-treatment size (cm)—mean ± SD	2.8 ± 1.1
*T stage—n(%)*	
T1	20 (76.9)
T2	6 (23.1)
*Histology—n(%)*	
Adenocarcinoma	16 (61.5)
Squamous	9 (34.6)
NSCLC NOS	1 (3.9)
Pre-treatment FEV_1_—mean ± SD	73.7 ± 16.4
Post-treatment FEV_1_—mean ± SD	75.1 ± 20.2
Change FEV_1_—mean ± SD	0.1 ± 13.5
Dose fractionation—n(%)	
54 Gy in 3 fractions	5 (19.2)
55 Gy in 5 fractions	15 (57.7)
60 Gy in 8 fractions	6 (23.1)
Surgery—n(%)	26 (100)
*Surgery type—n(%)*	
Lobectomy	18 (69.2)
Wedge resection	8 (30.8)
*Surgical approach—n(%)*	
VATS	21 (80.8)
VATS converted to open	3 (11.5)
Open	2 (7.7)

*NSCLC NOS* non-small cell lung cancer not otherwise specified; *FEV*_*1*_ forced expiratory volume in 1 s, *VATS* video-assisted thoracoscopic surgery

**Fig. 2:**
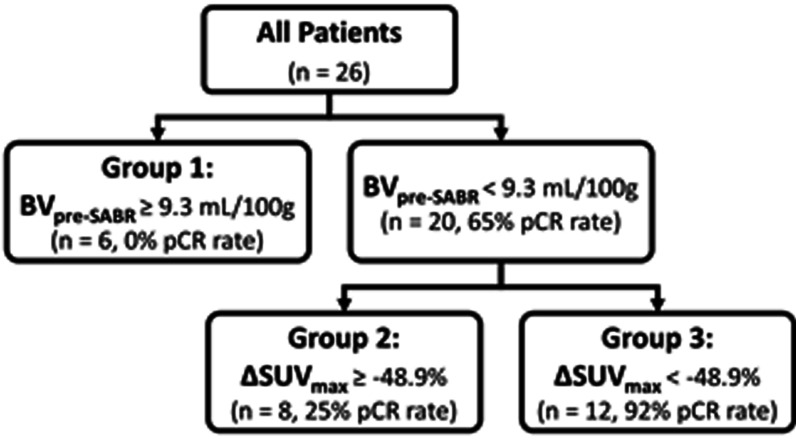
Recursive partitioning tree. *BV* blood volume, *SABR* stereotactic ablative radiation therapy, *pCR* pathologic complete response, *SUV* standardized uptake value

The RPA identified three patient groups based on tumour blood volume before SABR (BV_pre-SABR_) and change in SUV_max_ (ΔSUV_max_) as shown in Fig. 2. According to the RPA, group 1 was defined as baseline tumour blood volume (BV_pre-SABR_) ≥ 9.3 mL/100 g (n = 6, 0% pCR rate). No Group 1 patient showed pCR from the pathological analysis. All 6 patients had tumour residual disease after SABR treatment. Group 2 was defined by BV_pre-SABR_ < 9.3 mL/100 g and the percent change in SUV_max_ (ΔSUV_max_) ≥ − 48.9% after SABR (n = 8, 25% pCR rate). Group 3 was defined by BV_pre-SABR_ < 9.3 mL/100 g and ΔSUV_max_ < − 48.9% after SABR (n = 12, 92% pCR rate).

**Fig. 3:**
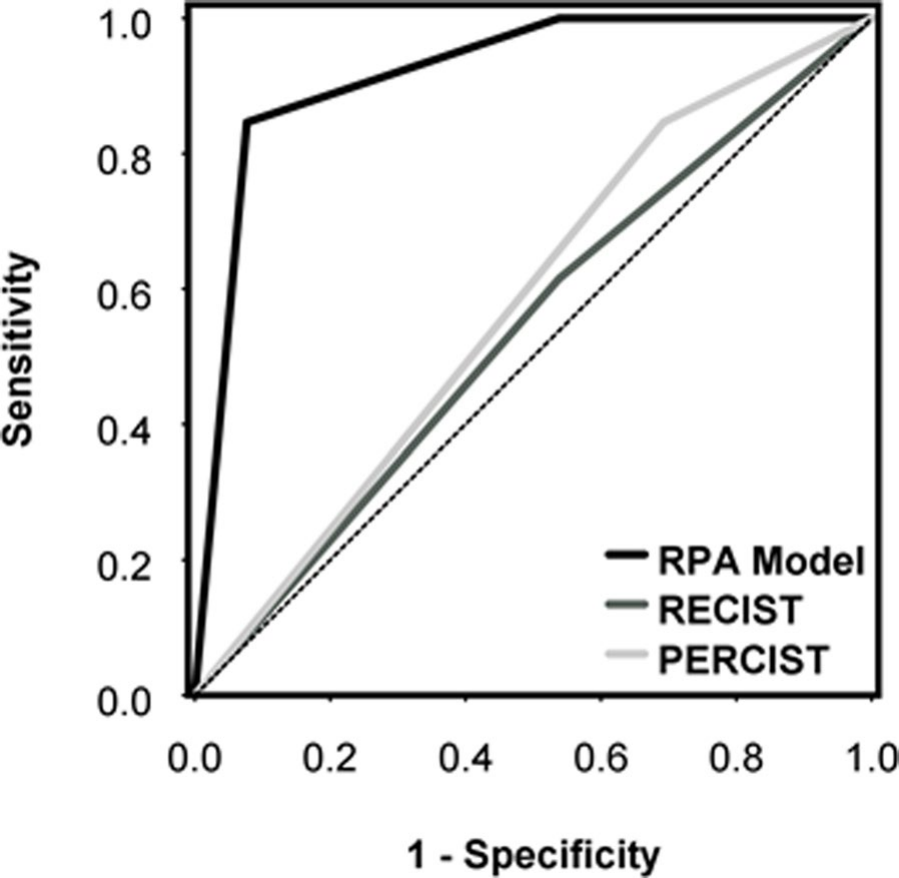
ROC curves. *RPA* recursive partitioning analysis, *RECIST* Response Evaluation Criteria in Solid Tumours 1.1, PERCIST Positron Emission Tomography Response Criteria in Solid Tumours 1.0

**Fig. 4:**
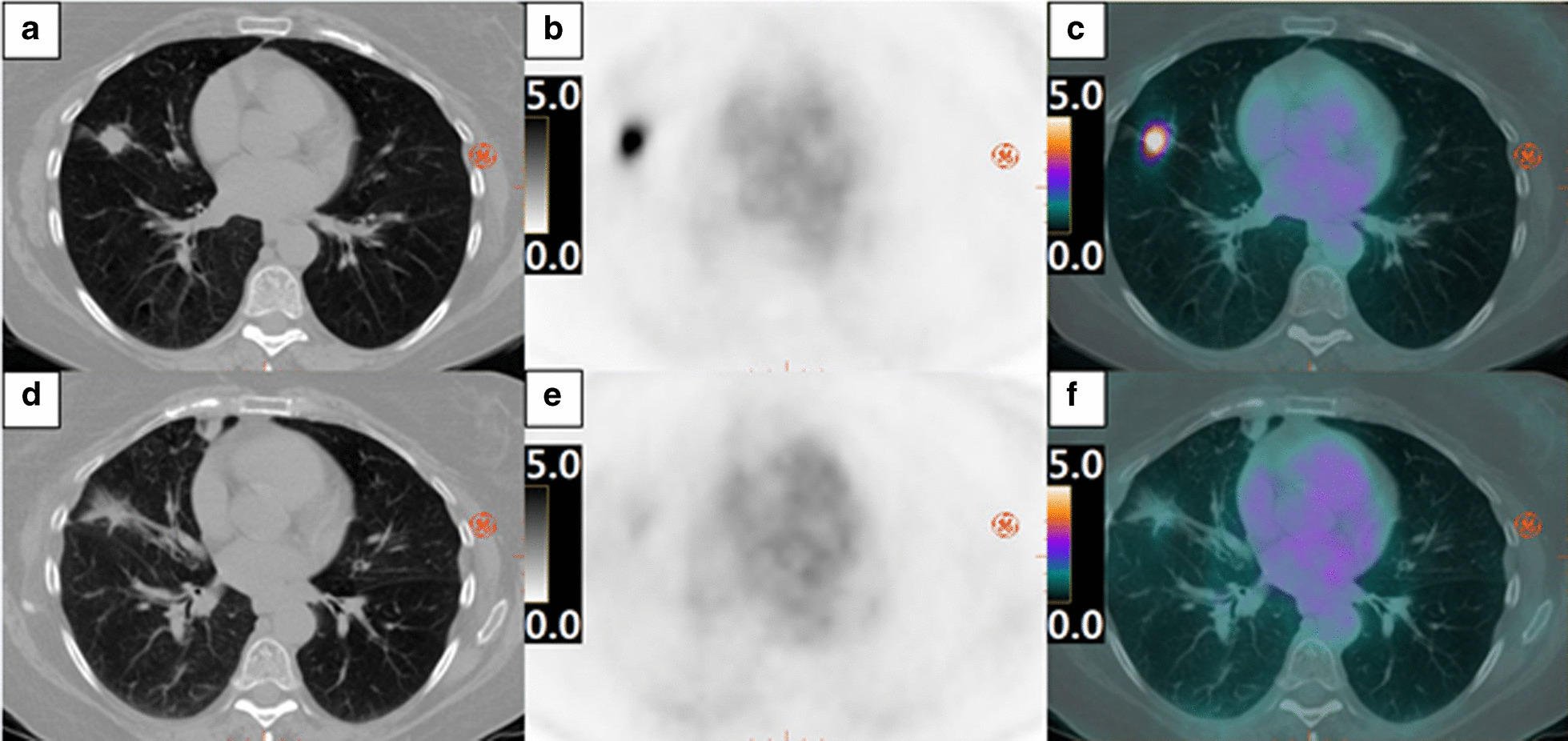
Example of RECIST Failure. Pre-SABR images of pCR patients on CT, PET SUV (range 0–5.0 g/mL), and PET/CT fused are shown in **a**, **b** and **c**, respectively. Post-SABR CT, PET and PET/CT fused images are shown in **d**, **e** and **f**, respectively. *RECIST* Response Evaluation Criteria in Solid Tumours 1.1, *SABR* stereotactic ablative radiation therapy, *pCR* pathologic complete response, *CT* computed tomography, *PET* positron emission tomography, *SUV* standardized uptake value

**Fig. 5:**
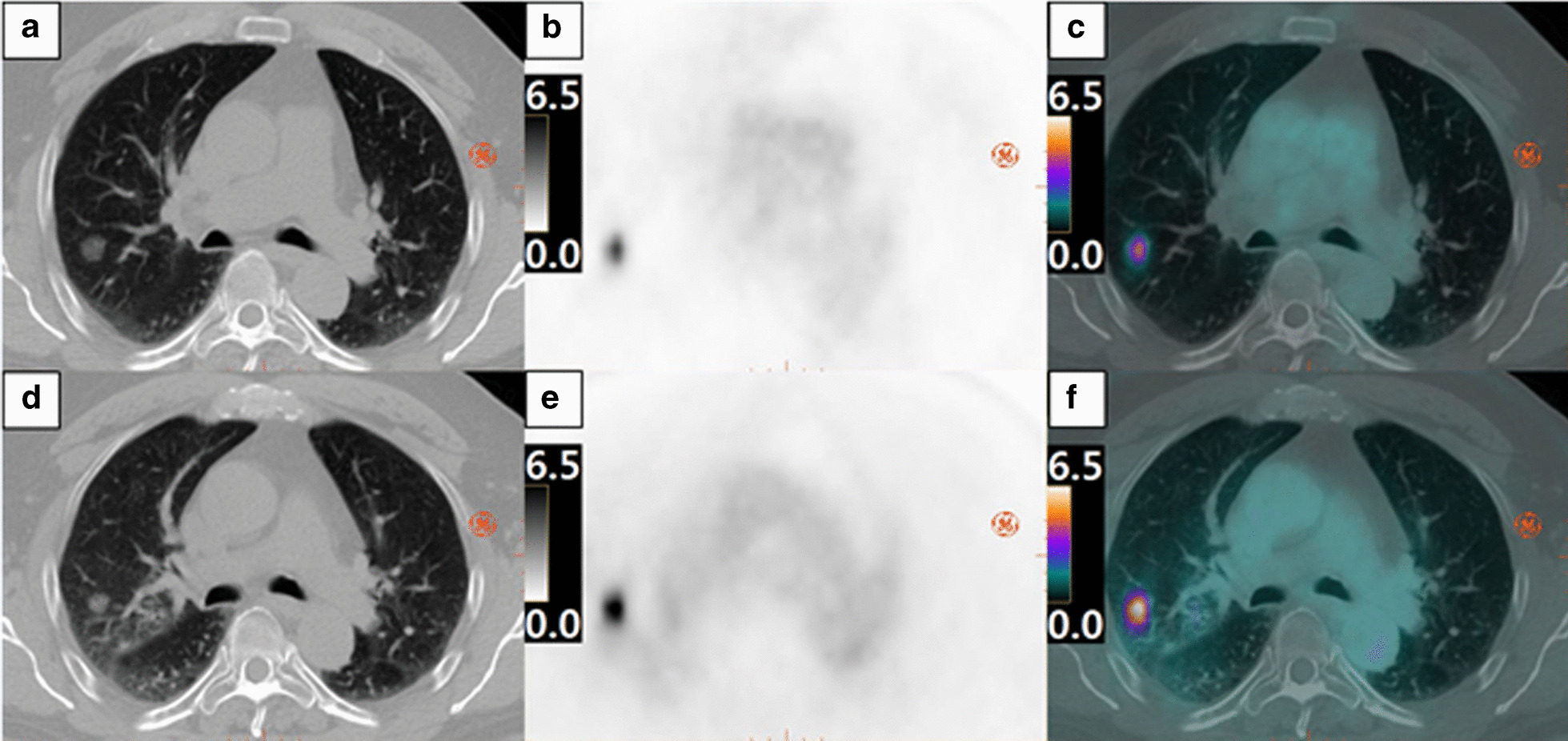
Example of PERCIST failure. Pre-SABR images of pCR patients on CT, PET SUV (range 0–6.5 g/mL), and PET/CT fused are shown in **a**, **b** and **c**, respectively. Post-SABR CT, PET and PET/CT fused images are shown in **d**, **e** and **f**, respectively. *PERCIST* PET Response Criteria in Solid Tumours, *SABR* stereotactic ablative radiation therapy, *pCR* pathologic complete response, *CT* computed tomography, *PET* positron emission tomography, *SUV* standardized uptake value

The RPA model was able to predict pCR with 85% sensitivity, 92% specificity, 92% PPV, 86% NPV, and concordance (area under the ROC curve) of 0.92 (95% confidence interval (CI) 0.82–1.00). In contrast, current clinical RECIST criteria of CR/PR applied to pre- and post-SABR from the same patient showed 46% sensitivity, 47% specificity, 38% PPV, 54% NPV, and concordance of 0.54 (95% CI 0.34–0.74) in predicting pCR. Furthermore, PERCIST criteria of CMR/PMR applied to pre- and post-SABR [^18^F]FDG scan showed 85% sensitivity, 31% specificity, 55% PPV, 67% NPV, and concordance of 0.58 (95% CI 0.35–0.80) in predicting pCR. DeLong test of the C-statistic showed that pCR prediction with RPA model was significantly different from RECIST and PERCIST (*p* < 0.01). RECIST vs. PERCIST for pCR prediction was not different (*p* = 0.81). ROC curves are shown in Fig. 3. Figure 4 shows increase in lesion diameter on CT and decrease in uptake of [^18^F]FDG in a patient with pCR while Fig. 5 shows the opposite in another patient with pCR-decreased diameter on CT and increased [^18^F]FDG uptake.

## Discussion

This study employed RPA decision trees to predict pCR after SABR in patients with early-stage NSCLC using biomarkers from [^18^F]FDG-PET and CTP. Our results suggest that pCR of early-stage NSCLC to SABR can be predicted with 85% sensitivity, 92% specificity, 92% PPV, and 86% NPV using biomarkers from [^18^F]FDG-PET and CTP study before and 8–10 weeks after treatment.

In comparison, RECIST and PERCIST criteria showed worse pCR prediction due to radiation induced lung injury (RILI) because recurrent tumour can show similar size and morphology change and [^18^F]FDG uptake as RILI. Examples from Figs. 4 and 5 may indicate why the RECIST and PERCIST criteria lack sensitivity, specificity, PPV and NPV in predicting pCR to SABR. To be effective in treating early-stage NSCLC, SABR requires better evaluation criteria for response than the current RECIST and/or PERCIST; our study suggests that combined [^18^F]FDG and CTP could be a viable alternative.

This study suggests that pCR to SABR could be predicted with the BV of NSCLC before and change in maximum uptake of [^18^F]FDG post-treatment. The predictive model using RPA decision trees separated SABR patients into three groups: Group 1: patients with BV_pre-SABR_ ≥ 9.3 mL/100 g; Group 2: patients with BV_pre-SABR_ < 9.3 mL/100 g and ΔSUV_max_ ≥ − 48.9%; and Group 3: BV_pre-SABR_ < 9.3 mL/100 g and ΔSUV_max_ < − 48.9%. In a previous study with NSCLC patients, [^18^F]FDG SUV_max_ and BV of NSCLC correlated with cell proliferation marker, Ki67 and with microvessel density marker, CD34 staining, respectively [11]. While angiogenesis is associated with microvessel density, it does not necessarily lead to high blood flow (delivery) because the increased interstitial fluid pressure (edema) from the immature and leaky neo-vessels may reduce blood flow, resulting in tissue hypoxia [12]. Therefore, our results are consistent with the notion that high BV may induce hypoxia thereby worsen the outcome of SABR while decrease in SUV_max_ is a key factor to predict the treatment outcome as it reflects slowdown of tumour proliferation.

Our study has several limitations. First was the small sample size. To address this limitation, we used RPA to identify imaging-based biomarkers that have potential for predicting pCR rather than the conventional logistic regression technique which requires larger sample sizes to improve model stability. [13, 14] Second limitation is that the PET image analysis was done without image registration to minimize blur from the motion of the tumour. Third limitations is that CT and PET for RECIST and PERCIST were not done with standard clinical protocol. CT was the average map of a CTP study done without breathhold, even though the images were co-registered before they were averaged together, there may still be residual motion blur. The PET SUV scan was acquired at 30 min post injection, the clinical SUV scan is normally acquired between 50 and 70 min post-injection. Fourth limitation is that pCR in some tumours might have been missed because the interval time (2 weeks) between completion of SABR and surgery could be too short. Nevertheless, we were able to create a predictive model based on imaging biomarkers from CTP and PET study that was significantly better than RECIST and PERCIST criteria; therefore, this RPA model warrants future external validation with larger sample size studies.

## Conclusions

In conclusion, this study shows that tumour BV before treatment (BV_pre-SABR_) and change in [^18^F]FDG SUV_max_ (ΔSUV_max_) at 8 weeks post-treatment can predict pCR of early-stage NSCLC to SABR with good sensitivity and high specificity. In comparison, RECIST and PERCIST criteria had poorer sensitivity and specificity in pCR prediction. While these findings were limited by the small sample size, the developed prediction model warrants further investigation.

## Data Availability

Support data is available to interested readers upon reasonable request to corresponding author.

## Abbreviations

[^18^F]FDG: Fluorine-18 fluorodeoxyglucose
BF: Blood flow
BV: Blood volume
CMR: Complete metabolic response
CR: Complete response
CT: Computed tomography
CTP: CT perfusion
ECOG: Eastern Cooperative Oncology Group
FEV_1_: Forced expiratory volume in 1 s
FOV: Field of view
K_1_: Influx rate constant
k_2_: Efflux rate constant
k_3_: Binding rate constant
k_4_: Dissociation rate constant
K_i_: Net Uptake/metabolic rate constant
MTT: Mean transit time
NPV: Negative predictive value
NSCLC: Non-small cell lung cancer
NOS: Not otherwise specified
pCR: Pathologic complete response
PERCIST: PET Response Criteria in Solid Tumours 1.0
PET: Positron emission tomography
PMR: Partial metabolic response
PPV: Positive predictive value
PR: Partial response
PS: Vessel permeability surface product
RECIST: Response Evaluation Criteria in Solid Tumours 1.1
RILI: Radiation induced lung injury
ROC: Receiver operating characteristic
RPA: Recursive partitioning analysis
SABR: Stereotactic ablative radiation therapy
SUV: Standardized uptake value
VATS: Video-assisted thoracoscopic surgery
